# Enhanced Differentiation of Three-Gene-Reprogrammed Induced Pluripotent Stem Cells into Adipocytes via Adenoviral-Mediated PGC-1α Overexpression

**DOI:** 10.3390/ijms12117554

**Published:** 2011-11-07

**Authors:** Pin-I Huang, Yueh-Ching Chou, Yuh-Lih Chang, Yueh Chien, Kuan-Hsuan Chen, Wen-Shin Song, Chi-Hsien Peng, Chin-Hong Chang, Shin-Da Lee, Kai-Hsi Lu, Yi-Jen Chen, Chia-Hua Kuo, Chuan-Chih Hsu, Hsin-Chen Lee, Ming-Chi Yung

**Affiliations:** 1 Institute of Clinical Medicine, National Yang-Ming University, Taipei 112, Taiwan; E-Mails: pinihuang@gmail.com (P.-I.H.); khchen3@vghtpe.gov.tw (K.-H.C.); chpeng1008@gmail.com (C.-H.P.); 2 School of Medicine, National Yang-Ming University, Taipei 112, Taiwan; E-Mails: ws.song@msa.hinet.net (W.-S.S.); lionel.lu@gmail.com (K.-H.L.); chenyj@vghtpe.gov.tw (Y.-J.C.); 3 Cancer center, Taipei Veterans General Hospital, Taipei 112, Taiwan; 4 Institute of Pharmacology, National Yang-Ming University, Taipei 112, Taiwan; E-Mails: ycchou@vghtpe.gov.tw (Y.-C.C.); ylchang@vghtpe.gov.tw (Y.-L.C.); g39005005@gmail.com (Y.C.); 5 College of Pharmacology, Taipei Medical University, Taipei 110, Taiwan; 6 Department of Pharmacy, Taipei Veterans General Hospital, Taipei 112, Taiwan; 7 Department of Medical Research and Education, Taipei Veterans General Hospital, Taipei 112, Taiwan; 8 Department of Surgery, Cheng-Hsin General Hospital, Taipei 112, Taiwan; 9 Shin Kong Wu Ho-Su Memorial Hospital, Taipei 111, Taiwan; 10 Department of Surgery, Chi-Mei Medical Center, Tainan 710, Taiwan; E-Mails: cmh7520@hotmail.com.tw (C.-H.C.); cchsu1967@hotmail.com (C.-C.H.); 11 Department of Physical Therapy, Graduate Institute of Rehabilitation Science, China Medical University, Taichung 404, Taiwan; E-Mail: shinda@mail.cmu.edu.tw; 12 Department of Medical Research and Education, Cheng-Hsin General Hospital, Taipei 112, Taiwan; 13 Laboratory of Exercise Biochemistry, Taipei Physical Education College, Taipei 111, Taiwan; E-Mail: kuochiahua@gmail.com; 14 Divison of Cardiovascular Surgery, Department of Surgery, Taiwan Adventist Hospital, Taipei 105, Taiwan

**Keywords:** induced pluripotent stem cell, c-Myc, peroxisome proliferator-activated receptor gamma coactivator-1α, brown adipocyte

## Abstract

Induced pluripotent stem cells formed by the introduction of only three factors, Oct4/Sox2/Klf4 (3-gene iPSCs), may provide a safer option for stem cell-based therapy than iPSCs conventionally introduced with four-gene iPSCs. Peroxisome proliferator-activated receptor gamma coactivator-1α (PGC-1α) plays an important role during brown fat development. However, the potential roles of PGC-1α in regulating mitochondrial biogenesis and the differentiation of iPSCs are still unclear. Here, we investigated the effects of adenovirus-mediated PGC-1α overexpression in 3-gene iPSCs. PGC-1α overexpression resulted in increased mitochondrial mass, reactive oxygen species production, and oxygen consumption. Microarray-based bioinformatics showed that the gene expression pattern of PGC-1α-overexpressing 3-gene iPSCs resembled the expression pattern observed in adipocytes. Furthermore, PGC-1α overexpression enhanced adipogenic differentiation and the expression of several brown fat markers, including uncoupling protein-1, cytochrome C, and nuclear respiratory factor-1, whereas it inhibited the expression of the white fat marker uncoupling protein-2. Furthermore, PGC-1α overexpression significantly suppressed osteogenic differentiation. These data demonstrate that PGC-1α directs the differentiation of 3-gene iPSCs into adipocyte-like cells with features of brown fat cells. This may provide a therapeutic strategy for the treatment of mitochondrial disorders and obesity.

## 1. Introduction

Stem cells have the potential for self-renewal and the ability to differentiate into many types of cells [1–3]. Recent techniques have demonstrated that induced pluripotent stem cells (iPSCs) can be generated from mouse embryonic fibroblasts (MEFs) and human fibroblasts via the retroviral transduction of four transcription factors: Oct-4, Sox2, Klf4 and c-Myc [4,5]. These cells are considered a promising resource for restorative cell therapy with a wide range of clinical applications for the treatment of different diseases. We recently demonstrated that iPSCs expressing only three factors, Oct4/Sox2/Klf4 (3-gene iPSCs), were capable of differentiating into hepatocyte-like cells, and the exclusion of the oncogene c-Myc resulted in decreased tumorigenicity [6]. These findings indicate that 3-gene iPSCs may be a safer alternative for stem cell-based therapy.

PGC-1α (peroxisome proliferator-activated receptor gamma coactivator-1α) was isolated from a brown fat library used to identify peroxisome proliferator-activated receptors γ (PPARγ)-interacting proteins. It regulates the expression of the uncoupling protein-1 (UCP-1) gene and several brown fat-selective genes during brown fat development [7]. This energy induced transcription coactivator is highly responsive to a variety of environmental cues, including temperature changes, nutritional status, and physical activity [8]. Multiple diseases caused by dysregulation of metabolism are known to be caused by aberrant PGC-1α expression [9]. However, the potential role of PGC-1α in regulating mitochondrial biogenesis and the differentiation of iPSCs is still unclear.

Because PGC-1α serves an important role in brown fat development, we aimed to determine if PGC-1α can mediate the differentiation of 3-gene iPSCs into adipose-specific lineages. In this study, we show that adenovirus-mediated PGC-1α overexpression led to increased mitochondrial mass and an increase in reactive oxygen species (ROS) production and O2 consumption. Microarray-based bioinformatics demonstrated that the gene expression pattern of PGC-1α-overexpressing 3-gene iPSCs resembled the expression pattern of adipocytes. Furthermore, PGC-1α overexpression enhanced adipogenic differentiation and the expression of several brown fat markers but suppressed osteogenic differentiation. These findings demonstrate an important role for PGC-1α in promoting the differentiation of 3-gene iPSCs into brown fat cells.

## 2. Results and Discussion

### 2.1. Characterization of 3-Gene iPSCs

**Figure 1:**
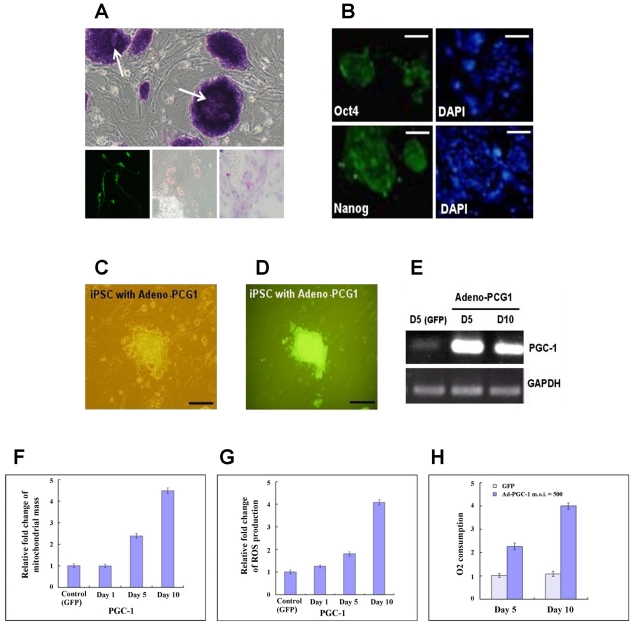
Characterization of 3-gene iPSCs overexpressing PGC-1α. (**A**) Upper: colonies of 3-gene iPSCs positive for alkaline phosphatase. Lower: differentiation of 3-gene iPSCs into ecto- (left), meso- (middle), and endo- (right) dermal lineages; (**B**) The iPSC colonies were positive for Oct-4 and Nanog; (**C**) Three-genes iPSCs were visualized using phase contrast microscopy; and (**D**) fluorescence microscopy 5 days after adenoviral infection; (**E**) RT-PCR analysis of PGC-1α expression in 3-gene iPSCs infected with the adenovirus expressing PGC-1α at 5 and 10 days post-transduction; (**F**) Relative changes in mitochondrial mass; (**G**) Relative changes in ROS production; (**H**) Relative oxygen consumption.

We used retroviruses to express the three reprogramming factors (Oct4/Sox2/Klf4) in MEFs, as previously described [6]. A resulting clone was positive for alkaline phosphatase expression (Figure 1A, upper) and the embryonic stem (ES) cell markers Oct-4 and Nanog (Figure 1B). After being treated with differentiation protocols specific for three separate dermal lineages, these 3-gene iPSCs could be differentiated into neuron-like cells (ectoderm), adipocyte-like cells (mesoderm) and hepatocyte-like cells (endoderm) (Figure 1A, lower). PGC-1α is considered a key molecule in regulating mitochondrial biogenesis and stem cell differentiation [7]. To investigate whether PGC-1α affects differentiation, we overexpressed PGC-1α in 3-gene iPSCs using an adenoviral system. iPSCs were efficiently infected with an adenovirus expressing GFP, and infection rates greater than 90% were routinely observed (Figure 1C and 1D). The expression of PGC-1α mRNA was consistently upregulated in ES cells 5 and 10 days after adenoviral transduction (Figure 1E).

### 2.2. Increased Mitochondrial Mass and Activity in PGC-1α-Overexpressing 3-Gene iPSCs

Previous data have shown that PGC-1α stimulates mitochondrial biogenesis, resulting in increased mitochondrial mass and respiration [10]. Therefore, we first investigated the changes in mitochondrial mass in 3-gene iPSCs overexpressing PGC-1α. The mitochondrial mass increased over time in PGC-1α-overexpressing cells compared to the controls (Figure 1F). Increased oxygen consumption is critical for PGC-1α-mediated mitochondrial activity during adipogenic differentiation. Therefore, we investigated the oxygen consumption of these cells using an oxygen-sensing electrode and measured the expression of reactive oxygen species (ROS) produced during mitochondrial biogenesis. PGC-1α significantly increased oxygen consumption 5 and 10 days post-transduction (Figure 1H). We also found a significant increase in ROS levels post-transduction (Figure 1G). These data indicate enhanced mitochondrial respiration in PGC-1α-overexpressing 3-gene iPSCs.

### 2.3. Genetic Profiling of 3-Gene iPSC Overexpressing PGC-1α

**Figure 2:**
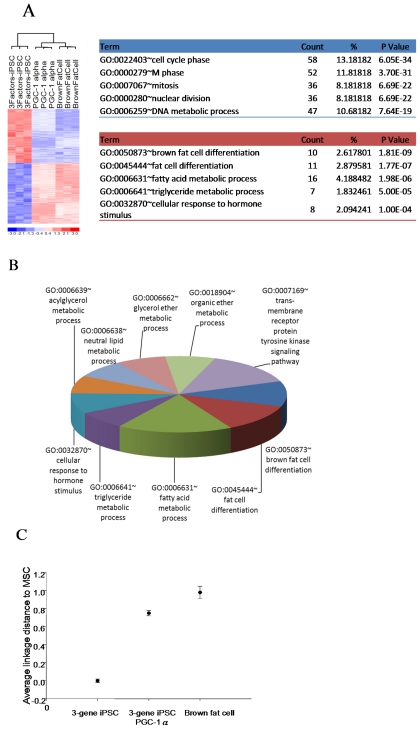
Gene expression profile of 3-gene iPSCs, PGC-1α-overexpressing 3-gene iPSCs and adipose tissue. (**A**) Gene expression microarray analysis showing genes that were differentially expressed among the three cell types using a hierarchy heat map; (**B**) Functional classification of the genes upregulated in PGC-1α-overexpressing 3-gene iPSCs compared to GFP-expressing 3-gene iPSCs, as determined by their Gene Ontology category; (**C**) Multidimensional Scaling further showed that the expression profile of PGC-1α-overexpressing 3-gene iPSCs was closer to the genetic signature of adipose tissue than the signature of 3-gene iPSCs.

We next analyzed the gene expression profile of PGC-1α-overexpressing 3-gene iPSCs using microarray analysis. The expression profiles were performed by Affymetrix Mouse Genome 430 2.0 Array, which contain 45101 probe sets selected from GenBank, dbEST, and RefSeq. Each gene may be detected by more than one probe. Using statistical methods to select 1000 probe IDs (*p*-value < 1E-10) which had the most significant expression difference, a total of 719 genes were differentially expressed in PGC-1α-overexpressing 3-gene iPSCs compared with GFP-expressing 3-gene iPSCs (Figure 2A). Furthermore, the average linkage distance analysis suggested that the gene expression profile of PGC-1α-overexpressing iPSCs was closer to the gene signature of adipose tissue than the expression profile of GFP-expressing 3-gene iPSCs (Figure 2C). Functional characterization of the significantly upregulated genes in PGC-1α-overexpressing 3-gene iPSCs during adipogenesis was performed using Gene Ontology. The processes associated with the upregulated genes and those that were represented significantly more than expected (*p* < 0.01), as identified by gene ontology, were primarily related to electron transport, cellular respiration, the generation of precursor metabolites and energy, the tricarboxylic acid cycle, fatty acid beta-oxidation, and carboxylic acid metabolic processes (Figure 2B). All of these pathways are necessary for mitochondrial function during adipogenic differentiation.

### 2.4. PGC-1α Enhances Adipogenesis and Inhibits Osteogenesis

**Figure 3:**
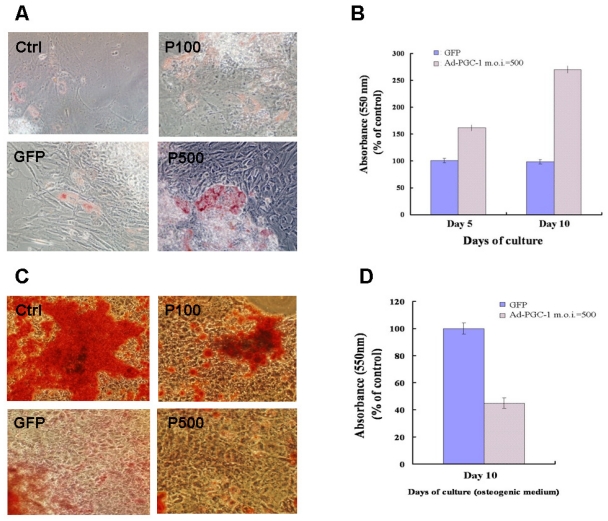
PGC-1α inhibits osteogenesis but enhances adipogenesis of 3-gene iPSCs. (**A**) Uninfected 3-gene iPSCs (as a control) or 3-gene iPSCs infected with an adenovirus expressing GFP (m.o.i. = 500) or PGC-1α (m.o.i. = 100/500) were cultured in adipogenic conditions for 10 days. After differentiation, the cells were stained with Oil Red O; and (**B**) staining was quantified using a spectrophotometer. The results are presented as the mean ± SE of three independent experiments; (**C**) Uninfected 3-gene iPSCs (as a control) or 3-gene iPSCs infected with an adenovirus expressing GFP (m.o.i. = 500) or PGC-1α (m.o.i. = 100/500) were cultured in osteogenic conditions for 10 days. After differentiation, the cells were stained with Alizarin Red; and (**D**) staining was quantified using a spectrophotometer. The results are expressed as the mean ± SE of three independent experiments.

Recently, an inverse relationship between adipogenesis and osteogenesis was shown in mesenchymal stem cells [2]. We further investigated whether PGC-1α overexpression modulates the differentiation of 3-gene iPSCs into adipocyte-like cells or osteocyte-like cells. As detected using Oil Red O staining, PGC-1α enhanced the adipogenic differentiation of iPSCs compared to the differentiation of GFP-expressing 3-gene iPSCs under adipogenic conditions (Figure 3A and 3B). Notably, PGC-1α expression also prevented the osteogenic differentiation of 3-gene iPSCs under osteogenic conditions, as detected using Alizarin Red staining (Figure 3C and 3D). These results suggest that PGC-1α enhances adipogenesis but prevents osteogenesis.

### 2.5. Upregulation of Brown Fat Markers in PGC-1α-Expressing 3-Gene iPSCs during Adipogenic Differentiation

**Figure 4:**
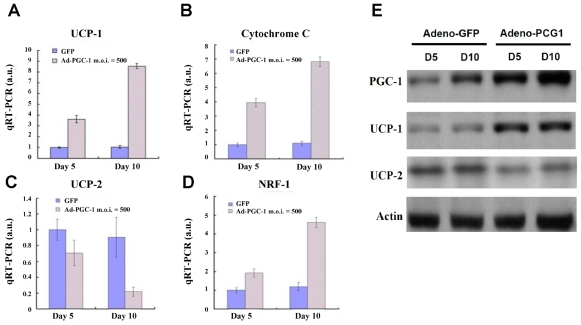
PGC-1α overexpression directs the differentiation of 3-gene iPSCs into brown adipocyte tissue during adipogenic induction. The mRNA level of (**A**) UCP-1; (**B**) cytochrome C; (**C**) UCP-2; and (**D**) NRF-1 was examined using qRT-PCR. The data are presented as the mean ± SD (*n* = 3); (**E**) The protein expression of PGC-1α, UCP-1 and UCP-2 was examined using western blotting. The data are presented as the mean ± SD (*n* = 3).

Two functionally different types of fat are present in mammals: white adipose tissue, which is the primary site for triglyceride storage, and brown adipose tissue, which is specialized for energy expenditure. To determine if PGC-1α-overexpressing 3-gene iPSCs preferentially differentiated into a specific type of adipose tissue, qRT-PCR was used to analyze the expression of adipose-specific markers. Adenovirus-mediated PGC-1α overexpression resulted in the upregulation of UCP-1, a brown fat marker, at day 5 post-differentiation. The upregulation of UCP-1 expression was further enhanced in cells overexpressing PGC-1α at day 10 post-differentiation (Figure 4A). In addition, we observed that PGC-1α significantly increased the expression of genes involved in mitochondrial function, including cytochrome C and nuclear respiratory factor-1 (NRF-1) (Figure 4B and 4D), in agreement with the mitochondria biogenesis data. In contrast, the expression of UCP-2, a white fat marker, was significantly reduced in PGC-1α-overexpressing 3-gene iPSCs compared to GFP-expressing cells (Figure 4C). Furthermore, the protein levels of these adipocyte-related markers were also assessed. Protein expression of PGC-1α and UCP-1 was significantly higher in PGC-1α-overexpressing 3-gene iPSCs than in control GFP-expressing 3-gene iPSCs at 5 and 10 days post-differentiation (Figure 4E). These findings reveal that PGC-1α directed the differentiation of 3-gene iPSCs into brown adipocytes, resulting in the upregulation of genes involved in brown fat differentiation and mitochondrial function.

### 2.6. Discussion

In this study, our data demonstrate that PGC-1α can promote the differentiation of 3-gene iPSCs into brown adipocytes with an accompanying increase in mitochondrial biogenesis and UCP-1 expression. In contrast, the white fat marker UCP-2 was downregulated following PGC-1α expression. Moreover, PGC-1α inhibited the differentiation of 3-gene iPSCs into osteocyte-like cells when cultured in osteogenic conditions. These results suggest that PGC-1α not only mediates mitochondrial biogenesis and respiration but is also involved in the development of brown adipose tissue.

Previous data have demonstrated that mice deficient in PGC-1α are cold sensitive and their brown fat tissue appears morphologically abnormal, with abundant accumulation of large lipid droplets, reminiscent of white adipose tissue [11]. The PGC-1α null mice also presented with a complex neurological disorder and neurodegeneration-associated hyperactivity [11,12]. These results suggest that genetic knockout of PGC-1α causes profound disturbances in energy homeostasis in addition to functional abnormalities in several tissues, including the liver, brown fat, brain, and heart [11]. Recent data using brown fat cells lacking PGC-1α demonstrated that there are significant defects in the ability of these cells to activate the program of gene expression linked to thermogenesis [13]. Uldry *et al*. also investigated whether the absence of PGC-1α can be compensated for by the presence of PGC-1β. Their data showed that a deficiency in either PGC-1α or β causes a small but significant decrease in mitochondrial gene expression. However, a deficiency in both PGC-1α and β causes a total loss of mitochondrial biogenesis and respiration [13]. This indicates that there is a strong requirement for either PGC-1α or β to maintain mitochondrial gene expression, density, and respiration. In the present study, our findings demonstrate that increased PGC-1α expression can increase mitochondrial mass, respiration and mitochondrial gene expression, including the expression of cytochrome C and NRF-1, and are consistent with previous findings.

iPSCs have been reported to differentiate into various lineages, including osteoblasts, chondrocytes, cardiomyocytes and adipocytes [6,14–16]. However, whether iPSC-derived adipocytes possess features of brown adipose tissue has not been examined. In addition, mitochondria play an important role the generation of heat by brown fat, and PGC-1 coactivators regulate several aspects of mitochondrial biogenesis and activity. The results of the Oil Red O staining indicate that PGC-1α enhanced adipogenesis under adipogenic conditions. In addition, we observed a significant increase in UCP-1 expression and a decrease in UCP-2 expression in PGC-1α-overexpressing 3-gene iPSCs-derived adipocytes (Figure 4A, 4C, and 4E). Other mitochondrial genes, such as cytochrome C and NRF-1, were upregulated in PGC-1α-overexpressing 3-gene iPSCs during adipogenic differentiation (Figure 4B and 4D). Moreover, microarray analysis indicated that the upregulated genes in PGC-1α- overexpressing 3-gene iPSCs are predominantly associated with the carboxylic acid metabolism, the generation of precursor metabolites and energy, electron transport, and fatty acid oxidation. These results suggest that PGC-1α is involved not only in thermogenesis but also in promoting the differentiation of 3-gene iPSCs into brown fat. However, a recent report has claimed that while PGC-1α is not required for brown fat differentiation, it does participate in differentiation-induced mitochondrial biogenesis [13]. This discrepancy could be due to the fact that Uldry *et al*. used immortalized preadipocytes for their experiments and that these cells have limited differentiation potential. To confirm whether PGC-1α is essential for adipose differentiation, it will be necessary to knockout PGC-1α to examine the effect of its loss on brown adipogenic differentiation. The predisposition toward brown fat adipogenesis in PGC-1α-overexpressing 3-gene iPSCs suggests that PGC-1α may determine the direction of differentiation for iPSCs during adipose development. It will be interesting to explore the upstream factors regulating the activity of PGC-1 activators and the downstream targets mediated by PGC-1α during brown fat differentiation. Future investigations are necessary to elucidate the relationship between PGC-1α and adipogenesis.

The incidence of *in vivo* teratoma formation has been a major unresolved problem for iPSC-based transplantation [14]. The c-Myc oncogene can contribute to tumorigenesis by over-stimulating cell growth and metabolism and/or by causing genomic instability. Deregulated expression of c-Myc occurs in a wide range of human cancers and is often associated with a poor prognosis, indicating a key role for this oncogene in tumor progression [17]. To eliminate teratoma formation, some alternative experimental approaches have been used in several studies. One recent study demonstrated that replacing c-Myc with L-Myc in combination with Oct4/Sox2/Klf4 promoted iPSC generation but not tumor formation [18]. Tsuji *et al*. demonstrated that transplantation of neurospheres derived from safe iPSC clones into injured spinal cords promoted functional recovery without teratoma formation [19]. Our recent study demonstrated that iPSCs expressing only three factors (Oct4/Sox2/Klf4) can provide a safer resource for stem cell-based therapy. The findings in the present study also suggest that 3-gene iPSCs may be an ideal platform for *in vitro* studies or drug screening.

## 3. Materials and Methods

### 3.1. iPSC Culture and Microarray Analysis

The iPSCs were reprogrammed via retroviral vectors expressing three transcription factors (Oct-4/Sox2/Klf4). iPSC culture, maintenance, and microarray analysis were conducted as described previously [6]. Briefly, total RNA was extracted from cells using Trizol (Life Technologies, Bethesda, MD, USA) and the Qiagen RNeasy (Qiagen, Valencia, CA, USA) column for purification. cRNA probe preparation, array hybridization and data analysis were performed according to Affymetrix^TM^ recommendations. Affymetrix^TM^ Mouse Genome 430 2.0 whole genome chips were used. RMA log expression units were calculated from Affymetrix GeneChip array data using the Bioconductor [20] software suite for the R statistical programming language [21]. The default RMA settings were used to background correct, normalize and summarize all expression values. Significant differences between sample groups were identified using the ‘limma’ package of Bioconductor. Briefly, a t-statistic was calculated as normal for each gene, and then a p-value was calculated using a modified permutation test. To control for multiple testing errors, a false discovery rate (FDR) algorithm was applied to these p-values to calculate a set of q-values, which are thresholds of the expected proportion of false positives, or false rejections, of the null hypothesis. A heat map was created using the dChip software [22]. Gene annotation and gene ontology were performed using the DAVID Bioinformatics Resources 6.7 interface[23].

### 3.2. Adenoviral Expression System

The adenovirus used in this study contains, in tandem, the green fluorescent protein (GFP) gene and the human PGC-1α cDNA downstream of separate cytomegalovirus promoters. Adenoviruses containing only GFP (Ad-GFP) or the antisense sequence of PGC-1α (Ad-AS-PGC-1α) were used as controls. Prior to adipocyte differentiation, 3-gene iPSCs were infected with Ad-GFP, Ad-AS-PGC-1α, or Ad-PGC-1α at a multiplicity of infection (m.o.i.) of 100 or 500 overnight. The medium was then changed to differentiation medium as described above. After the infected cells were cultured for 3 or 7 days, total RNA or total cell lysates were collected for the subsequent analysis of gene or protein expression, respectively.

### 3.3. Reverse Transcription Polymerase Chain Reaction (RT-PCR) and Quantitative RT-PCR

RT-PCR and qRT-PCR were used to determine the expression of PGC-1α. The RT-PCR and qRT-PCR were conducted as described previously [6]. The primer sequences used for RT-PCR are shown in Table 1.

### 3.4. Detection of Reactive Oxygen Species (ROS)

Production of ROS from 3-gene iPSCs was measured using hydroethidine (HE; Molecular Probes). Cells were incubated with 10 μM HE in culture medium for 60 min at 37 °C, washed, resuspended in 0.5 mL PBS and analyzed using flow cytometry.

### 3.5. Detection of Mitochondrial Mass

The mitochondrial mass was measured using the probe MitoTracker Red 580 (Invitrogen). Cells were incubated with 200 nM MitoTracker Red 580 in PBS for 15 min at 37 °C and then analyzed using flow cytometry.

### 3.6. Oxygen Consumption

The respiration of adipocytes was measured using an oxygen electrode (MT200/MT200A Respirometer Cell, Strathkelvin Instruments, North Lanarkshire, Scotland). PGC-1α- or GFP-expressing 3-gene iPSCs were trypsinized and rinsed with PBS. The cells were resuspended in DMEM-LG without supplements. Each sample (10^6^ cells) was analyzed during incubation in a magnetically stirred chamber over a period of 5 min at constant temperature (37 °C). The signals were detected and analyzed using software from Strathkelvin Instruments. The rate of oxygen consumption was normalized to the number of living cells, which was determined using Trypan Blue staining and counting cells using a hemocytometer.

### 3.7. Adipogenic Differentiation

Adipogenic induction was conducted as previously described [15]. iPSCs (1 × 10^5^) were cultured in adipogenic medium for 3 weeks. The adipogenic medium consisted of DMEM-LG supplemented with 10% FBS, 50 μg/mL ascorbate-2 phosphate, 100 nM dexamethasone, and 50 μM indomethacin (Sigma). For the evaluation of adipocytes, the cells were fixed with 4% formaldehyde and stained with Oil Red O (Sigma). Neurogenesis was assessed using the expression of nestin and other neural markers on days 7 and 14.

### 3.8. Osteogenic Differentiation

For osteogenic induction, iPSCs were cultured in DMEM-LG (Invitrogen) supplemented with 15% FBS, 50 μg/mL ascorbate-2-phosphate, 10 nmol/L dexamethasone, and 10 mmol/L β-glycerophosphate (Sigma, St. Louis, MO, USA) for 2 weeks. At the end of osteogenic induction, the cells were washed twice with PBS, fixed for 10 min at room temperature in 3.7% paraformaldehyde, and stained with von Kossa stain and Alizarin Red to assess osteogenic differentiation [1,2].

### 3.9. Statistical Analysis

All data are expressed as the mean ± standard deviation (SD). A one- or two-way ANOVA was used to determine the statistical significance of the differences. A *p* value less than 0.05 was considered significant. The statistics software used in this study was Sigma Stat 3.0.1 (SPSS, Chicago, IL, USA).

## 4. Conclusions

Mitochondrial dysfunction in adipocytes has been associated with obesity [24] and type 2 diabetes [25]. The major advantage of iPSCs over ES cells is that iPSCs can be derived from a patient’s own somatic cells, thereby avoiding immune rejection after transplantation and the ethical concerns raised by ES cells [4,5,26–28]. Factors that increase the PGC-1α level can be expected to drive iPSCs toward brown fat cells and mitochondrial biogenesis and respiration. BMP7 is a potential candidate that promotes brown adipose differentiation and thermogenesis via the upregulation of PGC-1α and other factors [29]. For iPSC-based treatment of obesity or mitochondrial disorders, it is plausible to contemplate the generation of patient-specific 3-gene iPSCs with high expression of PGC-1α. Transplantation of these iPSCs may provide a novel strategy for the treatment of these diseases. Future studies are required to verify the therapeutic potential of these strategies.

## Figures and Tables

**Table 1:** The sequences for the primers of RT-PCR.

Gene	Primer sequence
GAPDH	Forward:5′-CCC CAC ACA CAT GCA CTT ACC-3′ Reverse:5′-CCT ACT CCC AGG GCT TTG ATT-3′
PGC-1α	Forward:5-ATGCACTGACAGATGGAGACGTGAC-3′ Reverse:5-GTTCCTATACCATAGTCATGCATTG-3′
UCP-1	Forward:5′-TGGAATAGCGGCGTGCTTG-3′ Reverse:5′-CTCATCAGATTGGGAGTAG-3′
UCP-2	Forward:5′-TCTACAATGGGCTGGTTGC-3′ Reverse:5′-TGTATCTCGTCTTGACCAC-3′
NRF-1	Forward:5′-ACTGGAATTCCGTCGATGGTGAGA-3′ Reverse:5′-ACCTGACACAACACGGACAGAACT-3′
Cytochrome C	Forward:5′-ACGTGTCGACCTAATATGGGTGATGTTGAAAAAGG-3′ Reverse:5′-ACAGATCTTTCTCATTAGTAGCCTTTTTAAG-3′
